# Supplementary material for: REVersal of nEuromusculAr bLocking Agents in Patients Undergoing General Anaesthesia (REVEAL Study)
Source: J Clin Med. 2023 Jan 10;12(2):563. doi: 10.3390/jcm12020563 (PMC9866312; doi:10.3390/jcm12020563)
Supplement: Supplementary file 1 [file jcm-12-00563-s001.zip › jcm-2082755-supplementary.pdf]

Figure S1: (a) Missing data and random-forest-based imputation

This heatmap reports frequency of missing data, stratified by the primary outcome (type of NMBA reversal)

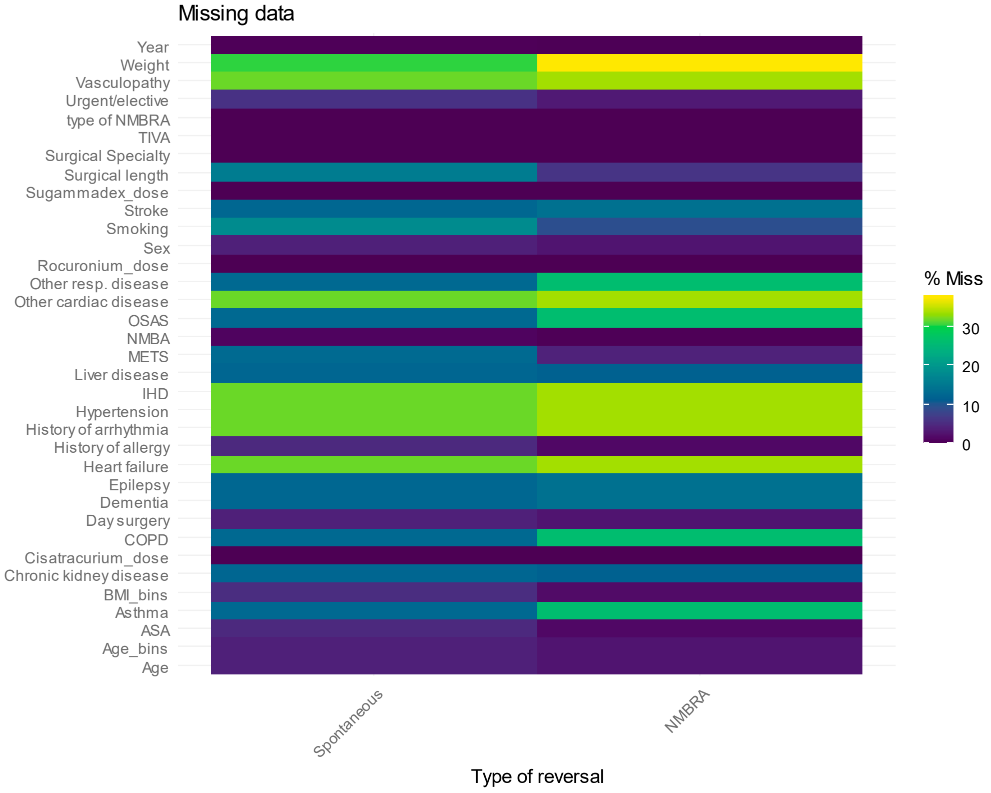

Figure S1 (b) Random forest performance

The following multiple plots report the results of random-forest-based imputation, with distribution of imputed data (light blue) compared to the original data (red)

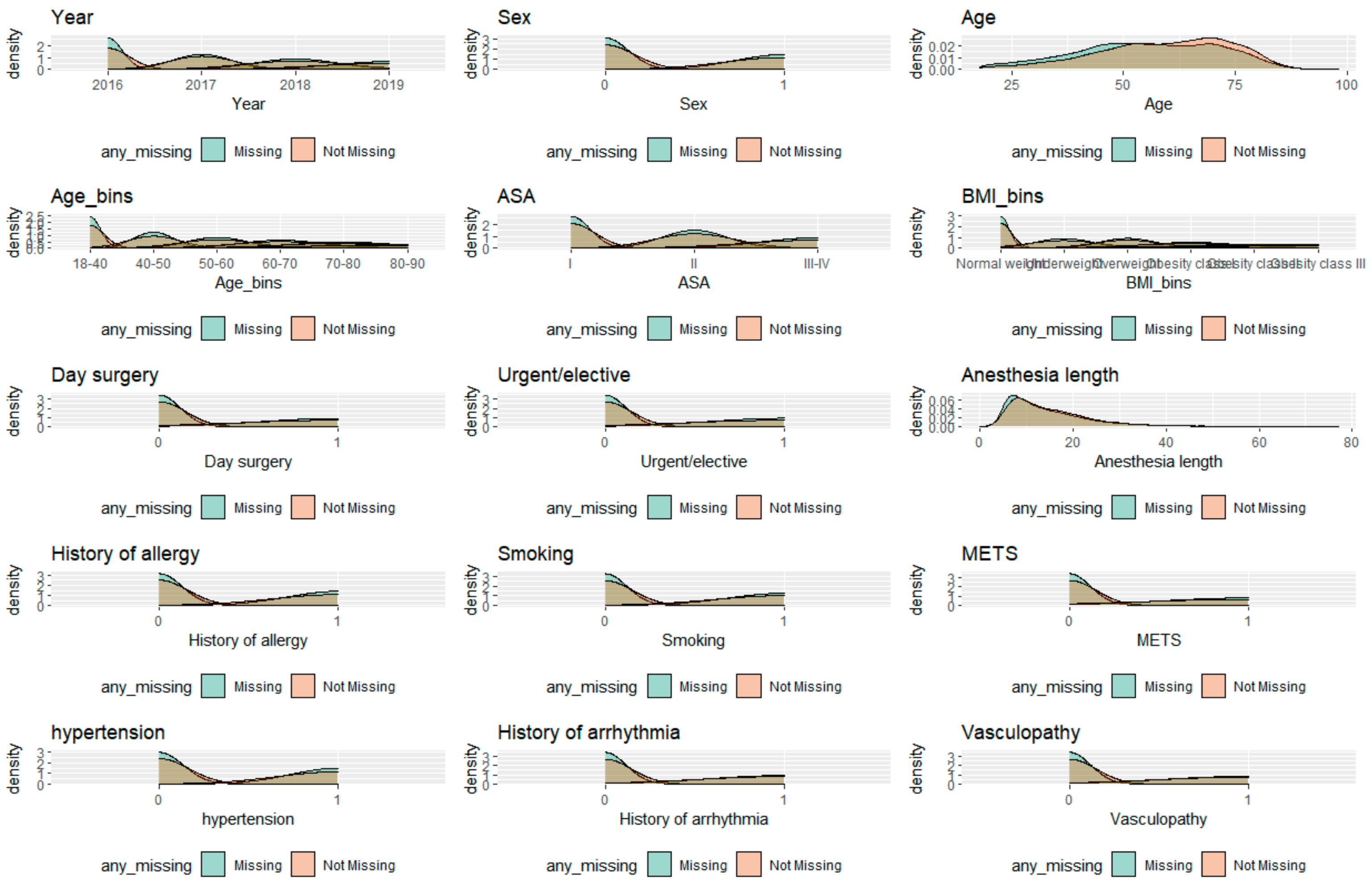

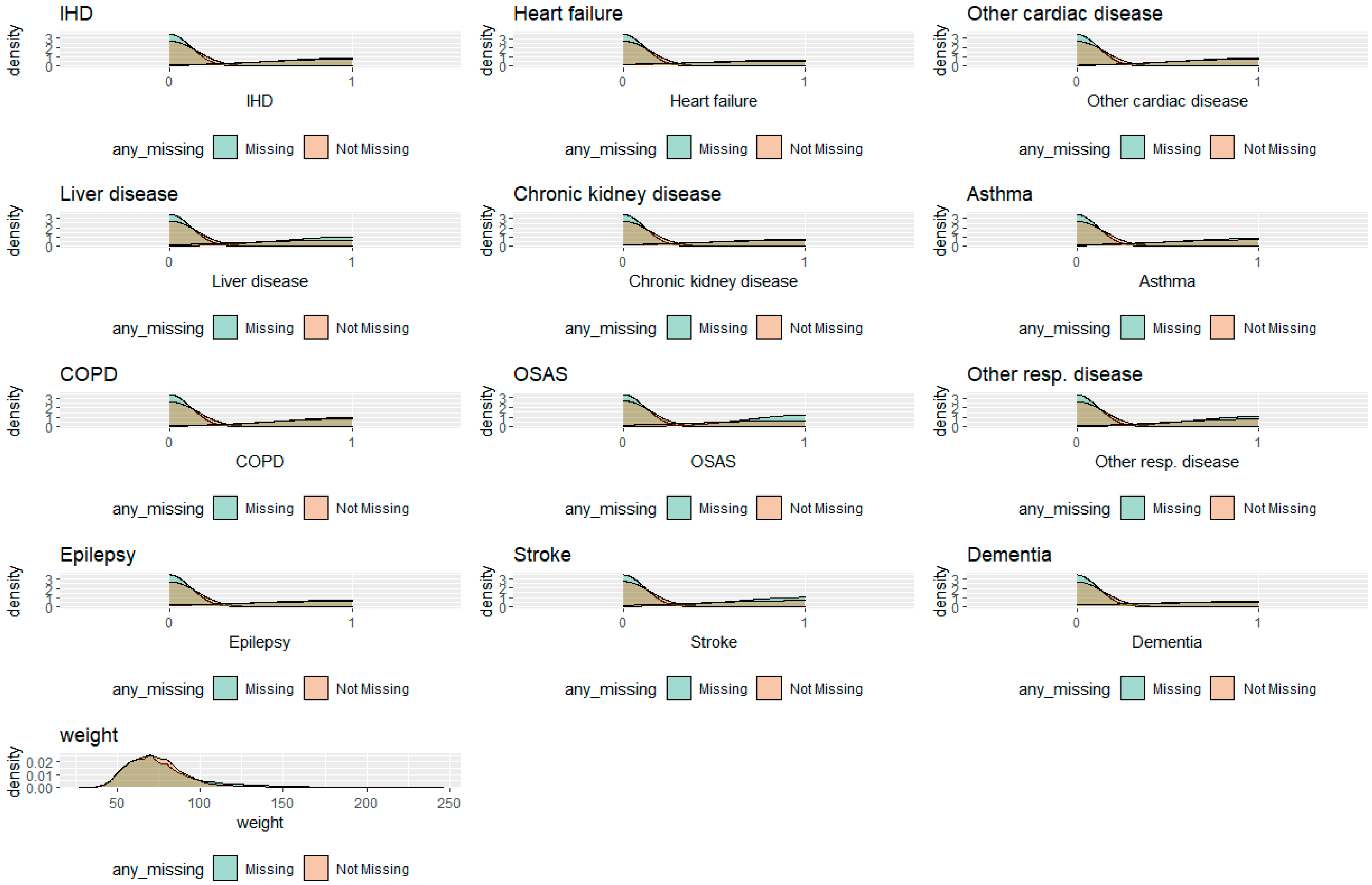
IHD
Heart failure
Other cardiac disease
Liver disease
Chronic kidney disease
Asthma
COPD
OSAS
Other resp. disease
Epilepsy
Stroke
Dementia
weight
density
0
1
0.00
0.01
0.02
50
100
150
200
250
any_missing
Missing
Not Missing

Figure S2: sensitivity analysis including non-missing data only

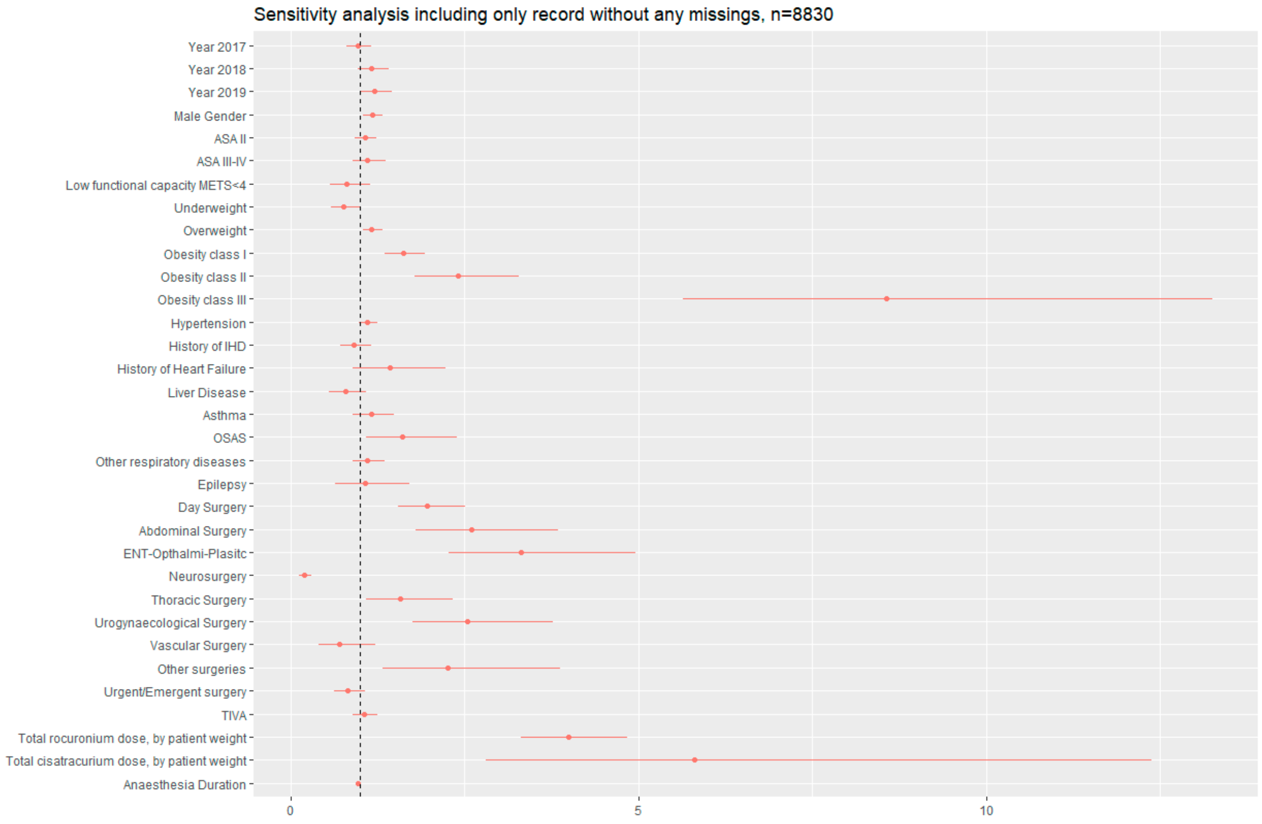

Table S1:Multivariable analysis on factors associated with sugammadex reversal in the subpopulation of patients paralyzed with rocuronium

Characteristic	OR[1]	95% CI[1]	p-value
Year (vs. 2016)			
2017	1.16	1.05-1.29	0.004
2018	1.49	1.36-1.65	<0.001
2019	1.47	1.34-1.62	<0.001
ASA (vs. class I)			
Class II	1.04	0.97-1.12	0.3
Class III-IV	1.53	1.38-1.71	<0.001
Low functional capacity (METS<4)	0.60	0.49-0.74	<0.001
BMI class (vs. normal weight)			
Underweight (<18.5)	0.87	0.75-1.00	0.05
Overweight (25-29.9)	1.16	1.08-1.24	<0.001
Obesity class I (30-34.9)	1.53	1.38-1.70	<0.001
Obesity class II (35-39.9)	3.52	3.01-4.13	<0.001
Obesity class III (>40)	11.8	9.77-14.3	<0.001
Day Surgery	1.25	1.11-1.40	<0.001
Type of surgery			
Orthopaedic surgery (ref)	—	—	
Abdominal surgery	3.49	2.91-4.22	<0.001
ENT- Ophthalmic -Plastic surgery	2.98	2.47-3.60	<0.001
Neurosurgery	0.12	0.10-0.15	<0.001
Thoracic surgery	1.75	1.45-2.11	<0.001
Urogynaecology surgery	2.67	2.21-3.25	<0.001
Vascular surgery	0.60	0.44-0.82	0.001
Other surgeries	2.23	1.77-2.84	<0.001
Urgent\Emergent surgery	0.63	0.54-0.73	<0.001
TIVA	0.93	0.86-1.01	0.077
Total rocuronium dose (on patient weight)	3.56	3.19-3.98	<0.001

Anaesthesia length (per 10-min increase)	0.95	0.94-0.95	<0.001
Allergy	0.95	0.89-1.01	0.11
Hypertension	1.08	1.02-1.15	0.013
History of heart failure	0.77	0.58-1.01	0.06
Liver disease	0.89	0.79-1.00	0.052
Asthma	1.24	1.07-1.44	0.005
OSAS	1.17	1.07-1.29	<0.001
Other respiratory comorbidities	1.15	1.02-1.30	0.025
Epilepsy	0.69	0.51-0.94	0.022

*TIVA=total intravenous anaesthesia, OSAS=Obstructive Sleep Apnoea Syndrome, IHD=Ischemic Heart Disease, MET=Metabolic equivalent of tasks

Figure S3: Forest plot multivariable regression of sugammadex vs neostigmine reversal in the subpopulation of patients paralyzed with rocuronium

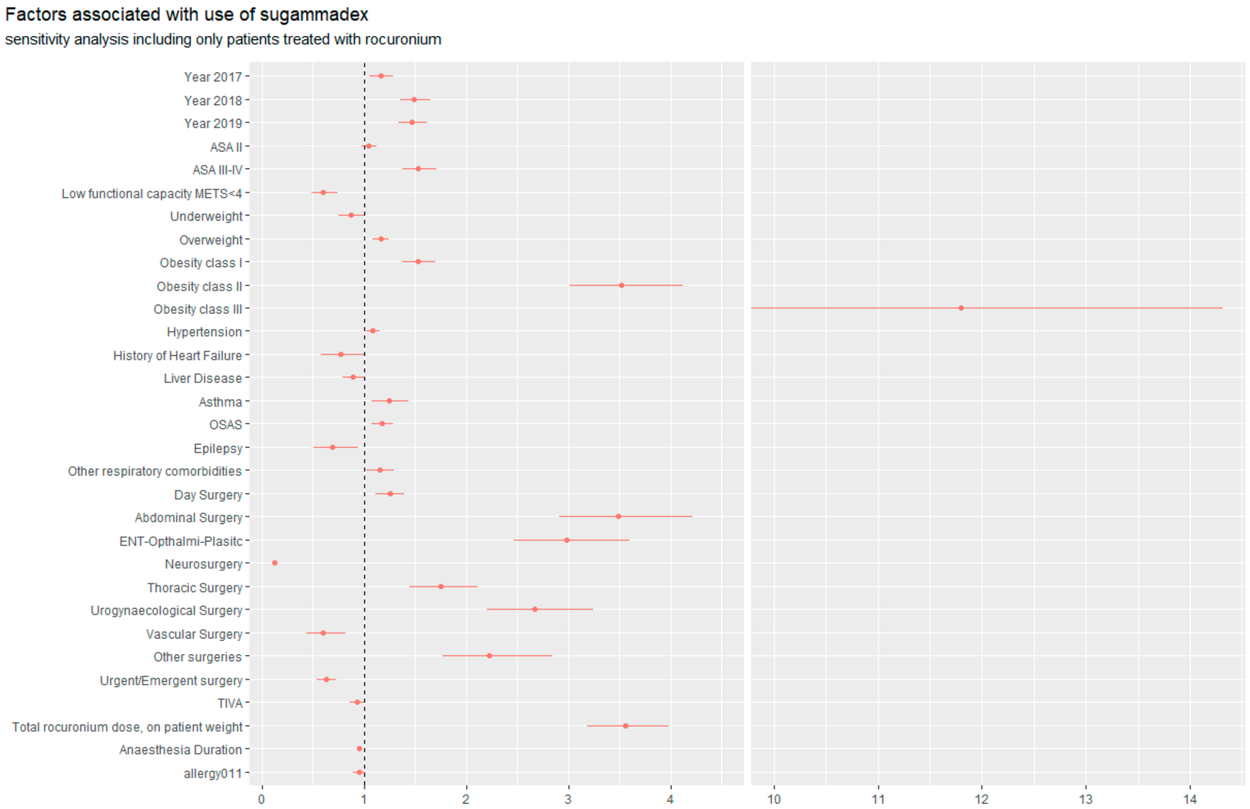

Figure S4

Total number of cases included (blue) by surgical specialty, and percent use of NMBA reversals in 2016 (light orange) vs. 2019 (dark orange),

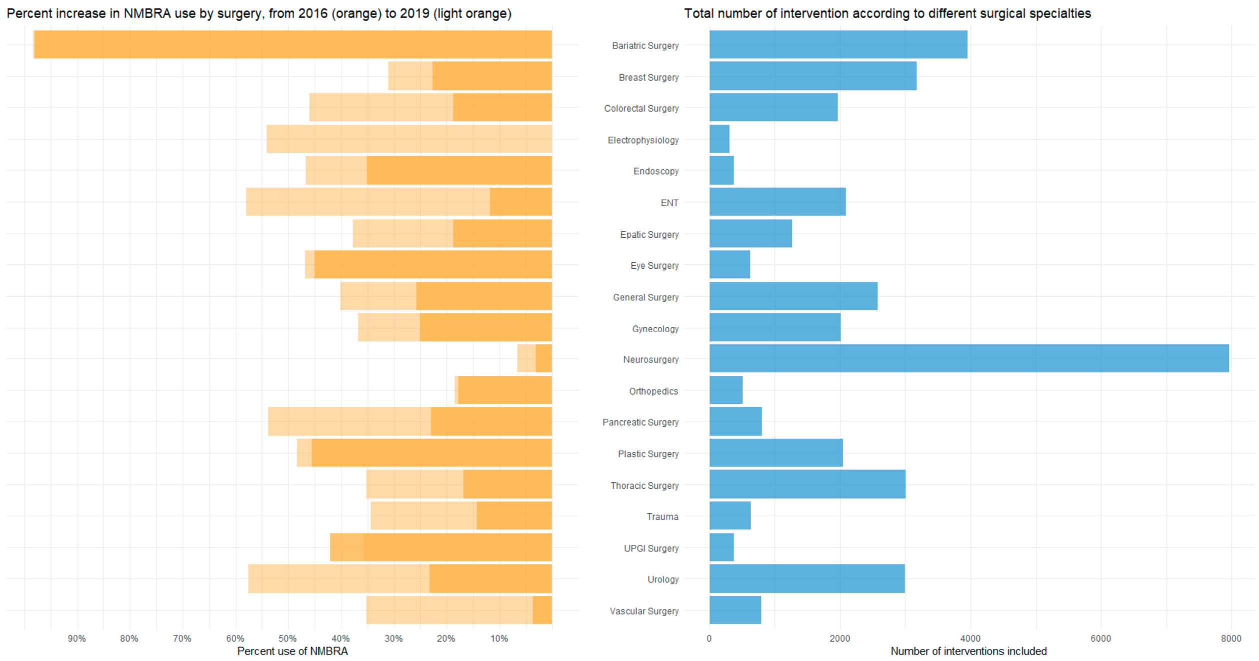

Figure S5 Performance analysis of the two regression models

	ROC	Sensibility	Specificity
NMBRA vs spontaneous reversal	0.82	0.90	0.49
Sugammadex reversal in rocuronium population	0.84	0.91	0.51

NMBRA vs spontaneous reversal model

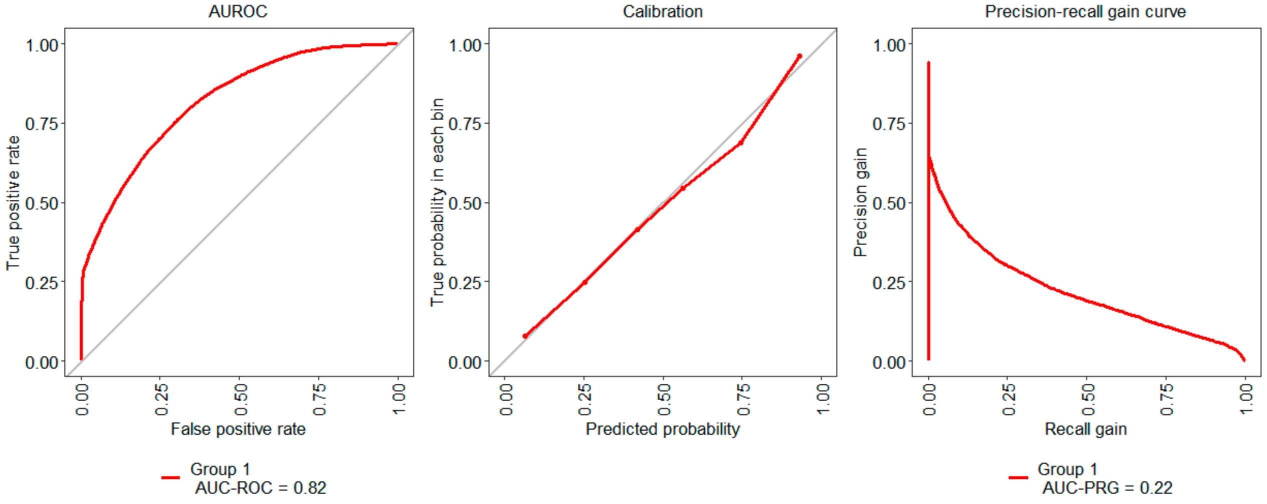

Sugammadex reversal in rocuronium population

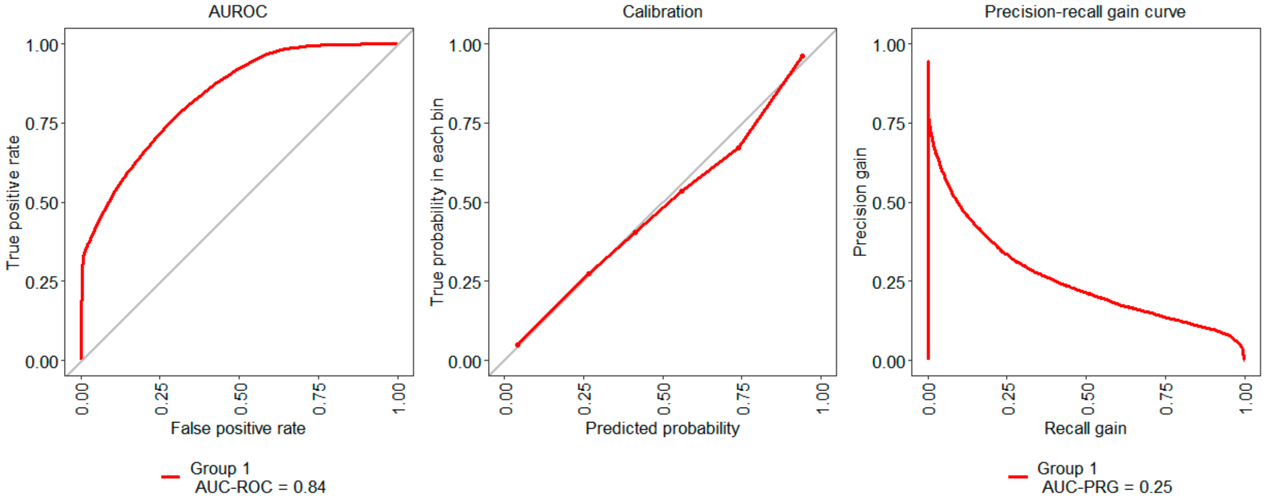

Table S2: time trends in NMBA reverersals in patients paralyzed with rocuronium

	2016			2017			2018			2019		
	Neostigmine	Sugammadex	Neither	Neostigmine	Sugammadex	Neither	Neostigmine	Sugammadex	Neither	Neostigmine	Sugammadex	Neither
N of surgeries	127 (2.6%)	1484 (30.4%)	3270 (67.0%)	157 (2.2%)	2389 (33.5%)	4575 (64.2%)	137 (1.5%)	3465 (38.4%)	5411 (60.0%)	310 (3.2%)	3967 (40.5%)	5520 (56.3%)
Age	54.9 (16.1)	48 (14.9)	56.4 (14.8)	55.9 (16.7)	50.7 (15.4)	56.9 (15.3)	58.4 (16.2)	53.1 (16.1)	57.2 (15.4)	59.2 (14.4)	52.9 (16.1)	57.2 (15.4)
Gender (male)	59 (3.4%)	400 (22.8%)	1299 (73.9%)	85 (3.1%)	738 (26.7%)	1942 (70.2%)	74 (1.9%)	1337 (34.8%)	2429 (63.3%)	165 (3.8%)	1714 (39.9%)	2417 (56.3%)
**Age bins:**18-40	22 (2.4%)	431 (47.8%)	448 (49.7%)	29 (2.3%)	589 (46.1%)	661 (51.7%)	22 (1.4%)	793 (49.2%)	798 (49.5%)	35 (2.0%)	934 (52.6%)	807 (45.4%)
40-50	23 (2.2%)	392 (37.4%)	632 (60.4%)	25 (1.7%)	615 (41.4%)	844 (56.9%)	21 (1.2%)	745 (43.5%)	947 (55.3%)	47 (2.5%)	825 (43.5%)	1023 (54.0%)
50-60	17 (1.8%)	276 (30.0%)	627 (68.2%)	29 (2.0%)	488 (34.2%)	911 (63.8%)	24 (1.2%)	732 (36.9%)	1226 (61.9%)	63 (3.0%)	857 (40.3%)	1204 (56.7%)
60-70	26 (3.0%)	157 (18.4%)	670 (78.5%)	30 (2.2%)	318 (23.2%)	1024 (74.6%)	27 (1.5%)	604 (33.3%)	1182 (65.2%)	91 (4.6%)	688 (34.9%)	1194 (60.5%)
70-80	20 (3.2%)	94 (15.2%)	506 (81.6%)	34 (3.2%)	222 (21.1%)	794 (75.6%)	36 (2.4%)	455 (30.7%)	989 (66.8%)	59 (3.8%)	504 (32.1%)	1008 (64.2%)
>80	1 (0.9%)	27 (23.5%)	87 (75.7%)	3 (1.4%)	63 (29.7%)	146 (68.9%)	5 (1.4%)	116 (33.0%)	231 (65.6%)	10 (2.6%)	134 (35.3%)	236 (62.1%)
**ASA Class:** ASA I	34 (2.9%)	253 (21.4%)	897 (75.8%)	49 (2.5%)	498 (25.3%)	1419 (72.2%)	42 (1.7%)	737 (30.2%)	1665 (68.1%)	79 (3.1%)	804 (31.3%)	1683 (65.6%)
ASA II	59 (3.0%)	477 (23.9%)	1458 (73.1%)	78 (2.5%)	843 (27.5%)	2148 (70.0%)	74 (1.8%)	1415 (34.9%)	2571 (63.3%)	186 (4.1%)	1505 (33.3%)	2823 (62.5%)
ASA III	17 (1.5%)	738 (66.6%)	353 (31.9%)	26 (1.4%)	1026 (53.8%)	856 (44.9%)	20 (0.9%)	1266 (54.0%)	1060 (45.2%)	36 (1.4%)	1580 (62.9%)	894 (35.6%)
ASA IV	0 (0.0%)	0 (0.0%)	5 (100.0%)	0 (0.0%)	6 (18.8%)	26 (81.2%)	0 (0.0%)	5 (19.2%)	21 (80.8%)	0 (0.0%)	21 (65.6%)	11 (34.4%)
**BMI Category** Underweight	0 (0.0%)	44 (35.2%)	81 (64.8%)	1 (0.4%)	84 (29.9%)	196 (69.8%)	6 (1.7%)	130 (37.2%)	213 (61.0%)	6 (1.8%)	108 (31.9%)	225 (66.4%)
Normal weight	56 (3.3%)	367 (21.5%)	1281 (75.2%)	71 (2.5%)	733 (25.3%)	2089 (72.2%)	64 (1.7%)	1224 (32.1%)	2525 (66.2%)	154 (3.8%)	1334 (32.9%)	2567 (63.3%)
Overweight	39 (3.2%)	238 (19.5%)	943 (77.3%)	55 (2.7%)	466 (22.7%)	1532 (74.6%)	48 (1.8%)	785 (29.7%)	1806 (68.4%)	99 (3.4%)	895 (31.1%)	1883 (65.5%)
Obesity class I	12 (3.0%)	99 (24.6%)	291 (72.4%)	22 (3.4%)	182 (27.8%)	451 (68.9%)	12 (1.4%)	268 (31.8%)	564 (66.8%)	31 (3.5%)	308 (35.2%)	537 (61.3%)
Obesity class II	3 (1.2%)	161 (66.0%)	80 (32.8%)	2 (0.6%)	240 (67.8%)	112 (31.6%)	5 (1.3%)	258 (66.0%)	128 (32.7%)	8 (1.8%)	322 (72.4%)	115 (25.8%)
Obesity class III	0 (0.0%)	557 (94.7%)	31 (5.3%)	2 (0.3%)	661 (92.7%)	50 (7.0%)	1 (0.1%)	744 (92.5%)	59 (7.3%)	1 (0.1%)	932 (94.7%)	51 (5.2%)
**Comorbidities** History of allergy	29 (2.0%)	545 (36.8%)	907 (61.2%)	52 (2.2%)	848 (35.4%)	1494 (62.4%)	41 (1.3%)	1207 (39.6%)	1799 (59.0%)	111 (3.5%)	1334 (41.8%)	1745 (54.7%)
Smoking	24 (2.4%)	369 (36.4%)	622 (61.3%)	32 (2.0%)	589 (36.1%)	1012 (62.0%)	36 (1.8%)	812 (39.9%)	1186 (58.3%)	56 (2.6%)	943 (43.6%)	1165 (53.8%)
Hypertension	37 (2.8%)	427 (32.6%)	846 (64.6%)	50 (2.2%)	724 (32.3%)	1470 (65.5%)	42 (1.5%)	1061 (37.1%)	1756 (61.4%)	108 (3.5%)	1236 (40.2%)	1729 (56.3%)
METS<4	0 (0.0%)	20 (29.4%)	48 (70.6%)	7 (3.2%)	44 (20.0%)	169 (76.8%)	4 (2.2%)	54 (29.0%)	128 (68.8%)	13 (6.9%)	79 (41.8%)	97 (51.3%)
Ischemic heart disease	3 (1.9%)	33 (21.0%)	121 (77.1%)	7 (2.4%)	80 (27.1%)	208 (70.5%)	12 (3.0%)	116 (29.0%)	272 (68.0%)	11 (3.0%)	147 (40.6%)	204 (56.4%)
History of arrhythmic disease	3 (1.9%)	37 (23.7%)	116 (74.4%)	5 (1.4%)	97 (27.2%)	255 (71.4%)	8 (1.6%)	159 (31.4%)	339 (67.0%)	16 (3.0%)	195 (36.3%)	326 (60.7%)

Vasculopathy	4 (5.6%)	19 (26.4%)	49 (68.1%)	2 (0.9%)	48 (22.2%)	166 (76.9%)	7 (2.2%)	70 (22.2%)	238 (75.6%)	8 (2.3%)	107 (30.4%)	237 (67.3%)
Heart failure	2 (5.7%)	15 (42.9%)	18 (51.4%)	1 (1.4%)	16 (22.2%)	55 (76.4%)	3 (2.5%)	47 (39.5%)	69 (58.0%)	3 (2.7%)	47 (42.0%)	62 (55.4%)
Other (Cardiac)	4 (2.3%)	66 (38.6%)	101 (59.1%)	8 (3.1%)	99 (38.8%)	148 (58.0%)	8 (1.8%)	152 (33.5%)	294 (64.8%)	19 (3.6%)	208 (39.1%)	305 (57.3%)
Asthma	2 (1.1%)	91 (50.8%)	86 (48.0%)	10 (4.4%)	107 (47.1%)	110 (48.5%)	5 (1.5%)	170 (50.6%)	161 (47.9%)	14 (3.6%)	178 (45.9%)	196 (50.5%)
COPD	7 (3.1%)	76 (33.6%)	143 (63.3%)	13 (2.8%)	119 (25.5%)	335 (71.7%)	13 (2.1%)	215 (35.1%)	385 (62.8%)	20 (3.0%)	228 (34.5%)	413 (62.5%)
OSAS	3 (2.2%)	109 (79.0%)	26 (18.8%)	2 (1.1%)	139 (76.4%)	41 (22.5%)	1 (0.4%)	170 (70.2%)	71 (29.3%)	3 (1.0%)	221 (73.9%)	75 (25.1%)
Other (Respiratory)	2 (0.7%)	167 (55.9%)	130 (43.5%)	2 (0.4%)	253 (55.1%)	204 (44.4%)	6 (0.9%)	324 (50.9%)	306 (48.1%)	15 (1.8%)	534 (62.5%)	306 (35.8%)
Renal failure	2 (4.3%)	12 (25.5%)	33 (70.2%)	3 (2.5%)	34 (28.1%)	84 (69.4%)	6 (3.0%)	68 (34.2%)	125 (62.8%)	8 (4.3%)	67 (35.6%)	113 (60.1%)
Liver disease	1 (2.0%)	12 (24.0%)	37 (74.0%)	5 (3.1%)	34 (20.9%)	124 (76.1%)	2 (1.0%)	37 (18.4%)	162 (80.6%)	5 (2.0%)	73 (28.6%)	177 (69.4%)
Epilepsy	1 (1.2%)	14 (16.9%)	68 (81.9%)	0 (0.0%)	21 (16.8%)	104 (83.2%)	1 (0.7%)	23 (16.2%)	118 (83.1%)	4 (3.2%)	22 (17.6%)	99 (79.2%)
Stroke	2 (2.4%)	26 (31.0%)	56 (66.7%)	6 (3.6%)	50 (29.9%)	111 (66.5%)	6 (3.7%)	48 (29.4%)	109 (66.9%)	6 (3.2%)	69 (36.7%)	113 (60.1%)
Dementia	5 (27.8%)	13 (72.2%)	18 (100.0%)	0 (0.0%)	7 (16.3%)	36 (83.7%)	1 (2.0%)	17 (33.3%)	33 (64.7%)	3 (6.0%)	13 (26.0%)	34 (68.0%)
Inpatients	101 (2.3%)	1304 (30.3%)	2898 (67.3%)	124 (1.9%)	2157 (32.9%)	4269 (65.2%)	99 (1.2%)	3197 (37.8%)	5170 (61.1%)	276 (3.0%)	3672 (40.2%)	5188 (56.8%)
Day Surgery	8 (5.2%)	73 (47.7%)	72 (47.1%)	26 (9.5%)	138 (50.2%)	111 (40.4%)	36 (7.4%)	248 (50.9%)	203 (41.7%)	29 (5.0%)	270 (46.3%)	284 (48.7%)
Abdominal Surgery	10 (0.8%)	956 (74.1%)	325 (25.2%)	16 (0.8%)	1291 (63.3%)	731 (35.9%)	16 (0.6%)	1559 (61.0%)	979 (38.3%)	75 (2.7%)	1825 (64.8%)	916 (32.5%)
ENT-Ophthalmic- Plastic	43 (6.7%)	242 (37.9%)	353 (55.3%)	44 (5.6%)	304 (38.7%)	437 (55.7%)	52 (4.1%)	629 (49.2%)	597 (46.7%)	35 (2.1%)	847 (51.7%)	755 (46.1%)
Neurosurgery	44 (2.5%)	6 (0.3%)	1679 (97.1%)	45 (2.4%)	26 (1.4%)	1792 (96.2%)	11 (0.5%)	68 (3.4%)	1935 (96.1%)	13 (0.6%)	119 (5.9%)	1891 (93.5%)
Orthopaedic	6 (4.8%)	21 (16.7%)	99 (78.6%)	18 (7.8%)	34 (14.7%)	180 (77.6%)	12 (5.2%)	54 (23.3%)	166 (71.6%)	11 (4.4%)	60 (24.1%)	178 (71.5%)
Other	0 (0.0%)	20 (27.4%)	53 (72.6%)	3 (1.8%)	73 (44.8%)	87 (53.4%)	1 (0.5%)	91 (46.0%)	106 (53.5%)	12 (3.9%)	121 (39.3%)	175 (56.8%)
Thoracic	10 (1.4%)	162 (22.3%)	556 (76.4%)	15 (1.3%)	402 (34.9%)	736 (63.8%)	25 (1.7%)	544 (36.1%)	940 (62.3%)	62 (4.1%)	486 (32.1%)	967 (63.8%)
Urogynaecology	12 (4.6%)	74 (28.6%)	173 (66.8%)	16 (2.2%)	244 (33.3%)	473 (64.5%)	9 (0.9%)	497 (47.0%)	551 (52.1%)	97 (8.6%)	471 (41.8%)	559 (49.6%)
Vascular	2 (5.4%)	3 (8.1%)	32 (86.5%)	0 (0.0%)	15 (9.7%)	139 (90.3%)	11 (6.4%)	23 (13.5%)	137 (80.1%)	5 (4.1%)	38 (31.1%)	79 (64.8%)
Elective	102 (2.5%)	1418 (34.5%)	2590 (63.0%)	145 (2.2%)	2249 (34.3%)	4169 (63.5%)	132 (1.6%)	3245 (38.5%)	5046 (59.9%)	279 (3.0%)	3719 (40.5%)	5178 (56.4%)
Urgent\emergent	7 (6.0%)	23 (19.7%)	87 (74.4%)	5 (1.7%)	61 (20.7%)	228 (77.6%)	3 (0.8%)	116 (32.8%)	235 (66.4%)	19 (5.6%)	121 (35.9%)	197 (58.5%)
TIVA	50 (5.7%)	214 (24.5%)	611 (69.8%)	58 (5.7%)	282 (27.8%)	674 (66.5%)	63 (4.4%)	419 (29.1%)	960 (66.6%)	72 (4.7%)	409 (27.0%)	1036 (68.3%)
Median dose pro kg (mg)	23±0.7 u	1.98 ±0.8 mg	-	21±0. 8 u	1.98±0.8 mg	-	25±1.2 u	2.24 (1.0)	-	0.25±0.9 u	2.26 (1.0)	-
Inhaled anaesthesia	77 (1.9%)	1270 (31.7%)	2659 (66.4%)	99 (1.6%)	2107 (34.5%)	3901 (63.9%)	74 (1.0%)	3046 (40.2%)	4451 (58.8%)	238 (2.9%)	3558 (43.0%)	4484 (54.2%)
Duration of surgery (mins)	121 (66)	107 (79.6)	137 (78.8)	143.6 (113)	118.1 (87.8)	152.7 (92.3)	158.2 (120.7)	129.2 (103.1)	156.6 (100.7)	190.9 (102.6)	133.8 (112.7)	152.4 (99.3)
